# In Situ-Forming Gels Loaded with Stimuli-Responsive Gated Mesoporous Silica Nanoparticles for Local Sustained Drug Delivery

**DOI:** 10.3390/pharmaceutics15041071

**Published:** 2023-03-26

**Authors:** Cristina de la Torre, Carmen Coll, Amelia Ultimo, Félix Sancenón, Ramón Martínez-Máñez, Eduardo Ruiz-Hernández

**Affiliations:** 1 Instituto Interuniversitario de Investigación de Reconocimiento Molecular y Desarrollo Tecnológico (IDM), Universitat Politècnica de València, Universitat de València, Camino de Vera s/n, 46022 Valencia, Spain; 2 CIBER de Bioingeniería, Biomateriales y Nanomedicina (CIBER-BBN), 28029 Madrid, Spain; 3 School of Pharmacy and Pharmaceutical Sciences, Trinity College Dublin (TCD), D02 W272 Dublin, Ireland; 4 Departamento de Química, Universitat Politècnica de València, Camino de Vera s/n, 46022 Valencia, Spain

**Keywords:** mesoporous silica, drug delivery, molecular gates, in situ-forming gels

## Abstract

A novel combination of in situ-forming hydrogels of hyaluronic acid with gated mesoporous materials was developed to design depots for local sustained release of chemotherapeutics. The depot consists of a hyaluronic-based gel loaded with redox-responsive mesoporous silica nanoparticles loaded with safranin O or doxorubicin and capped with polyethylene glycol chains containing a disulfide bond. The nanoparticles are able to deliver the payload in the presence of the reducing agent, glutathione (GSH), that promotes the cleavage of the disulfide bonds and the consequent pore opening and cargo delivery. Release studies and cellular assays demonstrated that the depot can successfully liberate the nanoparticles to the media and, subsequently, that the nanoparticles are internalized into the cells where the high concentration of GSH induces cargo delivery. When the nanoparticles were loaded with doxorubicin, a significant reduction in cell viability was observed. Our research opens the way to the development of new depots that enhance the local controlled release of chemotherapeutics by combining the tunable properties of hyaluronic gels with a wide range of gated materials.

## 1. Introduction

In recent years, the development of sustained and controlled drug release systems has gained increasing interest in the field of cancer treatment as a way to reduce side effects and to improve the availability of chemotherapeutics at the tumor site. In this scenario, mesoporous silica nanoparticles (MSNs) are attractive drug nanocarriers due to their high surface area, large pore volume, and tunable pore size, as well as their high efficiency in loading active molecules irrespective of their hydrophobic nature or relatively large size. Despite the significant potential of silica nanomaterials for healthcare applications, concerns related to their biocompatibility still remain to be fully addressed [1,2]. It is today well established that MSNs’ biocompatibility depends on their size, morphology, composition, surface functionalization, and administration route.

In terms of administration, in most studies nanocarriers are formulated as suspensions and injected. However, the incorporation of nanoparticles into other matrices can be of great interest for certain medical applications, such as for the development of local drug delivery systems for solid tumor treatment. For instance, Ranganath and colleagues developed a delivery system formed by an alginate gel matrix entrapping paclitaxel-loaded poly(lactic-co-glycolic acid) (PLGA) microspheres [3]. The in vitro release studies showed how the gel delivered the drug sustainably for more than 60 days with a minimal initial burst. Furthermore, following subcutaneous implantation in mice, the gel showed a more effective reduction in the tumor size when compared to the control group treated with Taxol, as the system reached the therapeutic concentration of paclitaxel for a longer period of time. Another example of hydrogel nanocomposite was developed by Arai and co-workers [4], who combined polymeric microspheres or liposomes loaded with doxorubicin (DOX) with a thermoreversible gelation polymer (TGP). Studies using U87MG, LN229, and G55 cell lines confirmed that TGP alone was non-toxic and that the liposomes and spheres in TGP were able to release DOX. In subsequent in vivo studies, the combination of microspheres or liposomes with TGP enhanced the antitumor activity and prolonged DOX release, increasing the efficacy of the treatment. In a different study, Ding and co-workers developed an injectable thermoresponsive hydrogel based on polyethylene glycol (PEG)-dipalmitoyl phosphatidylethanolamine (m-PEG-DPPE) and calcium phosphate nanoparticles for the local delivery of paclitaxel and temozolomide [5]. In vivo results confirmed that this approach also managed to effectively prolong the survival time in a rat model of glioma.

Similarly, some examples of MSNs being incorporated into a polymer matrix for release applications have been reported [6,7,8,9,10,11,12]. In the case of Zhu et al. [9], the incorporation of MSNs into a chitosan polymer to form a composite hydrogel was described. The authors found that the presence of gentamicin-loaded MSNs in the gel favored the sustained release of the drug over time, which did not occur when the gentamicin was directly loaded into the gel. Zuñiga and co-workers described the incorporation of rhodamine B (RhB)-loaded MSNs into a thermoresponsive poly(N-isopropylacrylamide) (PNIPAAm) hydrogel [10]. At temperatures higher than the lower critical solution temperature, the polymer chains collapsed and blocked the pores of the MSN materials, whereas at low temperatures the composite prevented the burst release that occurred when RhB was freely loaded into the polymer. Another example was developed by Zhao et al., who described a system based on ibuprofen-loaded MSNs embedded into a chitosan hydrogel to be used in titanium implants [11]. The mixture of MSNs and chitosan was deposited into a titanium plate, and the ibuprofen release profile was found to be responsive to pH changes and to the application of an electric field. Finally, Hu and co-workers incorporated MSNs loaded with bovine serum albumin into an alginate/hyaluronic/chitosan gel and successfully tested the composite in simulated gastrointestinal media [12].

The possibility of functionalizing the external surface of MSNs with molecular gates represents an exciting opportunity to build responsive composite materials. Such molecular gates are molecular or supramolecular ensembles that prevent cargo release until an external stimulus is applied. Gated mesoporous materials responsive to physical [13,14], chemical [15,16,17], and biochemical [18,19,20] stimuli have been reported, and a number of promising biomedical applications have been proposed based on the controlled release properties, proved both in vitro and in vivo, of these engineered nanoparticles [21,22,23,24,25,26,27,28].

Considering the multiple advantages that the incorporation of functionalized MSNs in a depot can provide in terms of controlling drug release, we report herein the preparation of composites formed by the combination of gated MSNs with hydrogels based on chitosan and hyaluronic acid. MSNs were loaded with a fluorescent dye (safranin O) or with a chemotherapeutic agent (DOX) and functionalized with disulfide-containing PEG chains acting as pore gatekeepers, which allows drug release to be triggered by the presence of reducing agents. This approach paves the way to developing multiple combinations for the application of this technology, employing different gated materials able to respond to distinct external stimuli from gels acting as local depots.

## 2. Materials and Methods

### 2.1. Chemicals

The chemicals tetraethyl orthosilicate (TEOS), n-cetyltrimethylammonium bromide (CTAB), sodium hydroxide (NaOH), safranin O, glutathione (GSH), (3-mercaptopropyl) trimethoxysilane (MPTMS), 2,2′-dipyridyl disulfide, peroxidase from horseradish (HRP), H_2_O_2_ 30%, β-glycerophosphate, and poly(ethylene glycol) methyl ether thiol (Mn 800) were provided by Aldrich. Doxorubicin hydrochloride (DOX) was supplied by Sequoia Research Products. For cell culture studies, U271 malignant glioma cells, Eagle’s minimum essential medium (MEM), penicillin–streptomycin, phosphate-buffered saline (PBS), fetal bovine serum (FBS), glutamine, trypsin, and cell proliferation reagent (WST-1) were obtained from Sigma-Aldrich. Tyramine-derivatized hyaluronic acid was purchased from Contipro A.S. Ultra-pure chitosan (UP CL214, Protasan) was supplied from Pronova Biomedical (Norway). The analytical-grade solvents were provided from Scharlab (Barcelona, Spain). All reagents were used as received.

### 2.2. General Techniques

The synthesized nanomaterials were characterized using standard techniques. Powder X-ray diffraction (PXRD) measurements were carried out using a Bruker AXN D8 Advance diffractometer using Cu-Kα radiation. Thermogravimetric analysis (TGA) was performed with a TGA/SDTA 851e (Mettler Toledo), using an oxidant atmosphere (air, 80 mL/min) with a heating ramp of 10 K per minute from 293 K to 1273 K, followed by an isothermal heating step at this temperature for 30 min. Transmission electron microscopy (TEM) micrographs were taken with a 100kV JEOL JEM-1010 Electron Microscope. Nanoparticle tracking analysis (NTA) was performed using a Nanosight NS300 instrument, while dynamic light scattering (DLS) measurements were carried out with a Zetasizer Nano analyzer, both from Malvern. N_2_ adsorption–desorption isotherms were recorded on a Micromeritics TriStar II Plus automated surface area and porosity analyzer, after degassing the samples at 120 °C in vacuum overnight. The adsorption data were then used to calculate specific surface areas and pore size using Brunauer, Emmett, and Teller (BET) and Barret, Joyner, and Halenda (BJH) models, respectively. Elemental analysis (EA) was performed with a CE Instrument EA-1110 CHN Elemental Analyzer. Fluorescence measurements were carried out with a JASCO FP-8500 Fluorescence Spectrophotometer. A DMI8 inverted laser scanning confocal microscope ((Leica Microsystems, Wetzlar, Germany) using a 40x oil objective was used for cell studies.

### 2.3. Synthesis of Mesoporous Silica Nanoparticles (MSNs)

Mesoporous silica nanoparticles were synthesized as reported previously [29,30]. Firstly, CTAB (1.00 g, 2.74 mmol), used as a surfactant, was dissolved in 480 mL of distilled H_2_O. Then, NaOH (3.5 mL, 2M) in distilled H_2_O was added to the solution, while the temperature was raised to 80 °C. TEOS (5 mL, 2.57 × 10^−2^ mol) was added dropwise to the mixture, which was subsequently stirred for 2 h. The final white precipitate was collected by centrifugation, washed with distilled H_2_O and ethanol, and dried at 60 °C (MSNs as synthesized). To remove the template and obtain the final porous material (MSNs), the solid was calcined at 550 °C using an oxidant atmosphere for 5 h.

### 2.4. Synthesis of S1 and S2

Materials S1 and S2 were synthesized as in a previous study of the group [31]. Briefly, safranin O dye (140.34 mg, 0.40 mmol) was added to a suspension of calcined MSNs (500 mg) in acetonitrile (15 mL). The mixture was sonicated for 10 min and stirred for 24 h at room temperature. The obtained material S0 was filtered off and dried under vacuum. Then, S0 (150 mg) was resuspended in acetonitrile (8.5 mL) in the presence of an excess of safranin O to avoid early dye release from the mesopores due to the concentration gradient, and MPTMS (464.4 μL, 2.5 mmol) was added, stirring the mixture for 5.5 h at room temperature. Afterwards, 2,2′-dipyridyl disulfide (550.8 mg, 2.5 mmol) was added, and the reaction was left to stir for an additional 12 h at room temperature. The resulting material, S1, was filtered off and dried under vacuum. In the last step of functionalization, S1 (100 mg) and poly(ethylene glycol)methyl ether thiol (0.5 mmol) were suspended in acetonitrile (3.33 mL) in the presence of an excess of safranin O. After 12 h of stirring, the final material S2 was isolated by centrifugation, washed with water, and dried under vacuum.

### 2.5. Synthesis of S3

S3 was synthesized with a similar method used to obtain solid S2. In this case, the chemotherapeutic drug doxorubicin (DOX) was employed as pore cargo instead of the dye. Calcined MSNs (200 mg) and DOX (100 mg, 0.17 mmol) were suspended in distilled H_2_O (7 mL). The final material S3 was isolated by centrifugation, washed with water, and dried under vacuum.

### 2.6. Synthesis of In Situ-Forming Gels

For the preparation of hyaluronic-acid-based gels (HA gels), a precursor solution of tyramine-derivatized hyaluronic acid in PBS was prepared at 2% *w*/*v* under stirring overnight at room temperature. A stock solution of H_2_O_2_ was prepared by adding 33 µL of H_2_O_2_ 30% in 10 mL of PBS. A stock solution of peroxidase from horseradish (HRP) was prepared by dissolving 0.856 mg of HRP in 10 mL of PBS. We used 2 separate vials to prepare a final gel volume of 4 mL. In vial A, 120 µL of H_2_O_2_ solution was added, and in vial B 120 µL of HRP solution (60 µL/mL gel). After stirring for two minutes, the corresponding nanoparticles were suspended in vial B and mixed gently using a pipette. Finally, a two-way syringe was used to quickly mix solutions A and B into a gel mold. The gels were then formed by the enzymatic oxidative coupling of the tyramine moieties in HA [32]. A few minutes later, 0.5 mL HA gels containing the nanoparticles were removed from the mold.

Chitosan-based gels were prepared as previously described [33]. Briefly, ultra-pure chitosan (80 mg) was dissolved in cold distilled H_2_O at pH 8–9. Next, 280 mg of β-glycerophosphate (β-GP) in distilled water at the same pH was added dropwise to the chitosan solution on ice. The corresponding nanoparticles were then added, and the final dispersion was gently mixed. Stable composite gels with a final volume of 0.5 mL were formed upon heating to 37 °C.

### 2.7. Cargo Release Studies

To provide a qualitative confirmation of the gating abilities of the synthesized S2 and S3 materials, cargo release assays were performed employing the reducing agent GSH as a release stimulus. In order to do that, 0.5 mg of each solid was suspended in 1.5 mL of distilled H_2_O at pH 7.5 with or without GSH (10 mM) and stirred. Several aliquots were taken at different time points and centrifuged. The supernatant was then analyzed to monitor the dye (S2) or drug (S3) release through the emission band of safranin O or doxorubicin centered at 585 nm (λ_ex_ = 520 nm) or 557 nm (λ_ex_ = 495 nm), respectively.

For release studies from the gels, 500 µL of HA gel with 6 mg/mL of S2 or S3 were placed into a well in a 12-well plate (3 mg of nanoparticles/gel). Then, 2 mL of PBS with GSH at final concentration of 10 mM were added. As a control, the same experiment was carried out in the absence of GSH. The plate was left in an incubator at 37 °C under moderate stirring. Aliquots of 250 µL were taken and the same volume was replaced with fresh solution. After centrifugation, the fluorescence of safranin O or DOX supernatants was measured in a 200 µL cuvette.

### 2.8. Nanoparticle Tracking Analysis (NTA)

The concentration of nanoparticles released from the gels was monitored using a Nanosight NS300. NTA was used to visualize and analyze the size of nanoparticle dispersions, by attending to their rate of Brownian motion, in the 10 nm–2000 nm diameter range in liquids. Determining factors for the analysis were the viscosity of the liquid, temperature, and size of the particle. Due to the light that the sample’s particles scatter when exposed to a laser, the particles can be detected. The light scattered was captured by digital photography, and the motion of each particle was tracked from frame to frame. Each aliquot from the gel release studies was diluted using distilled H_2_O and measured in the recommended concentration range (106–109 particles/mL). This operation was repeated three times.

Moreover, in order to confirm the integrity of S2 and S3 nanoparticles after being released from the gels, 0.5 mL of hyaluronic-acid-based gel with 6 mg/mL of nanoparticles were added into a vial with PBS (pH = 7), which was placed in a water bath at 37 °C under slight continuous stirring. Then, 1 mL aliquots were taken and replaced by fresh media once a day for 10 consecutive days. The mean size and size distribution of the nanoparticles released in the solution were measured by DLS and observed by TEM.

### 2.9. Cell Culture Conditions

The U271 cells were routinely grown in Eagle’s MEM supplemented with 10% fetal bovine serum (FBS), 1% penicillin–streptomycin, and 10% glutamine, at 37 °C in an atmosphere of 5% CO_2_ and underwent passage twice a week.

### 2.10. WST-1 Cell Viability Assay

Cells were cultured in sterile 24-well microtiter plates at a seeding density of 1.5 × 10^4^ cells/well and were allowed to settle for 24 h. Further, 0.5 mL of HA gels were placed in cell culture inserts with a polyethylene terephthalate (PET) track-etched membrane of 8.0 μm pore size, which allowed the passage of the nanoparticles (as verified by NTA). The inserts were removed at given time points (24, 48, 72 h). After 2 washes with PBS, 30 μL of WST-1 reagent (from a 50 mg/mL stock) was added to each well, and the plates were incubated for 1 h. Following thorough shaking for 1 min, absorbance was measured at 450 nm using a reference wavelength of 690 nm. Doxorubicin in sterile water was added to the wells as positive control.

### 2.11. Live Confocal Microscopy of Cells Treated with MSN-Loaded Gels

U271 cells were seeded in 24 mm glass coverslips in 24-well microtiter plates at a seeding density of 1.5 × 10^4^ cells/well. After overnight incubation, cells were treated with 0.5 mL HA gel containing 3 mg of the corresponding nanoparticles (i.e., S2 or S3) using cell culture inserts as mentioned before. After 24 h, the insert and the medium were removed, and cells were washed with PBS. Then, cells were fixed using paraformaldehyde 4% and visualized under a confocal microscope. The fluorescent signal of nuclear staining dye 4′,6-diamidino-2-phenylindole and DOX or safranin O inside the U271 cells was examined by a DMI8 inverted laser scanning confocal microscope. All confocal images were acquired using the same settings. Identical experiments were performed three times to check reproducibility.

## 3. Results and Discussion

### 3.1. Design and Synthesis of Gated MSNs

**Figure 1:**
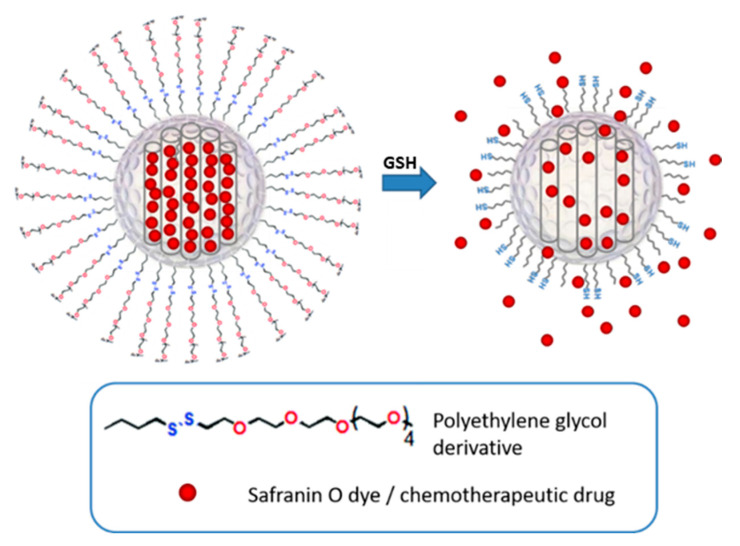
Schematic representation of the gated material (S2 and S3) capped with PEG chains via disulfide linkage, and its activation with intracellular GSH.

MSNs represent an ideal inorganic matrix that has a number of desirable features for the development of efficient drug delivery systems. Among these, it is important to mention MSNs’ chemical inertness and high and homogeneous porosity, the broad options of functionalization they offer, and their very high storage capacity for molecules of interest. With the aim of developing a gated stimuli-responsive device, we chose to employ PEG chains (Mn 800) with a terminal disulfide group attached to the silica surface as the capping system (see Figure 1), envisioning that it would respond to the presence of GSH in the cells. Therefore, using CTAB as a structure-directing agent and TEOS as a silica precursor, we obtained the starting mesoporous inorganic nanoparticles. From this base material, and following the loading and functionalization processes described above, we obtained the materials S2, loaded with safranin O, and S3, loaded with doxorubicin.

### 3.2. Characterization of the Materials

**Figure 2:**
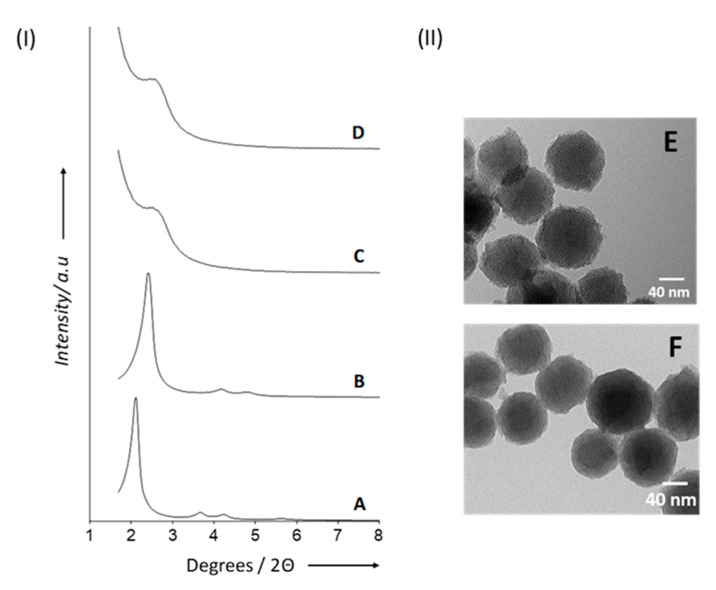
(**I**) Powder X-ray diffraction patterns of as-synthesized MSNs (**A**), calcined MSNs (**B**), solid S1 (**C**), and solid S2 (**D**). (**II**) TEM images of solids S2 (**E**) and S3 (**F**).

Once we obtained the described materials, they were characterized using several techniques. Figure 2I shows the powder X-ray diffraction (PXRD) patterns of the materials at different stages of synthesis and functionalization. For the as-synthesized MSNs (Figure 2(IA)), the characteristic four low-angle reflections attributed to a typical mesoporous hexagonal array indexed as (100), (110), (200), and (210) Bragg peaks can be observed. In Figure 2(IB), instead, it can be appreciated how calcination induced a significant shift in the (100) and a slight broadening of the (110) and (200) reflections. We calculated an a0 (cell parameter) of 40.2 Å for the calcined MSNs. For solids S1 (Figure 2(IC)) and S2 (Figure 2(ID)), the decreased scattering contrast following the loading/functionalization procedure determined the loss of the (110) and (200) reflections. Nevertheless, the presence of the (100) peak in their PXRD patterns shows that the mesoporous matrix was not significantly altered. The TEM micrographs shown in Figure 2II also confirm the mesoporous nature of materials S2 and S3, together with their spherical morphology (80–100 nm diameter). Additionally, DLS studies revealed mean hydrodynamic diameters of 87.0 nm for calcined MSNs, 122.5 nm for S2, and 140.2 nm for S3 (Figure S1).

From the N_2_ adsorption–desorption analysis, applying the BJH and BET models on the adsorption branch of the isotherm, we calculated a pore diameter of 3.2 nm, a pore volume of 0.91 cm^3^ g^−1^, and a specific surface area of 919.6 m^2^ g^−1^ for the starting calcined material. Looking at the isotherms obtained from its analysis (Figure S2), an adsorption step at P/P0 values between 0.1 and 0.3 can be observed, according to a type IV isotherm typical of mesoporous systems. Moreover, at high relative pressures (P/P0 > 0.8), a distinctive H1 hysteresis loop indicates that the large gaps between the nanoparticles have been filled. For the solids S2 and S3, instead, the N_2_ adsorption–desorption isotherm is typical of mesoporous materials after the loading of the mesopores. Table 1 shows the BET specific surface values, pore volumes, and pore sizes, as calculated from the N_2_ absorption–desorption isotherms.

Moreover, the contents of grafted and loaded molecules in solids S2 and S3 were determined by thermogravimetric and elemental analysis. Table 2 summarizes the most relevant results obtained for the different materials.

### 3.3. Functional Redox-Responsive Controlled Release

**Figure 3:**
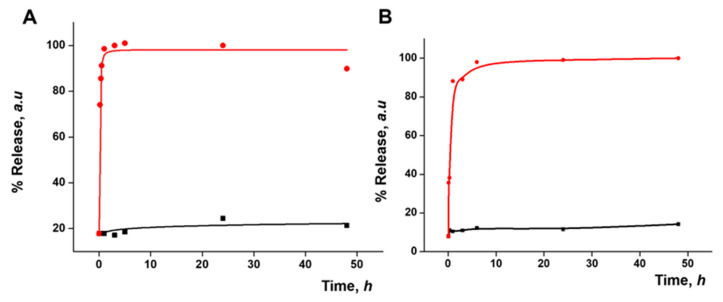
Kinetic release profile of safranin O delivered from S2 (**A**) or of DOX delivered from S3 (**B**) in water in the absence (black) and in the presence (red) of GSH (10 mM).

Dye release studies were carried out with both S2 and S3 to test the gating mechanism. The disulfide bonds in the PEG chains on the nanoparticle surface were expected to be cleaved by a reducing medium. In a typical experiment, a suspension of the material in water at pH 7.5 was prepared, with and without the addition of GSH (10 mM) as a release stimulus. This concentration of GSH was chosen considering that normal intercellular GSH levels are 1–10 mM and that in tumor cells they can even increase up to 2–10 times [34,35]. At given time points, aliquots were taken and centrifuged to remove the nanoparticles. The release was monitored by measuring the fluorescence signal of the safranin O fluorophore or of the DOX at 585 nm (λ_ex_ = 520 nm) and 557 nm (λ_ex_ = 495 nm), respectively (Figure 3). An initial release, up to 20% of the maximum release, was observed, likely due to the residue of cargo molecules on the surface of the nanoparticles. In both cases, a poor release was seen in the absence of the stimulus, while the presence of GSH triggered a fast delivery of the loaded cargo, achieving 90% of the maximum release within a few minutes.

### 3.4. Design and Characterization of In Situ-Forming Gels

**Figure 4:**
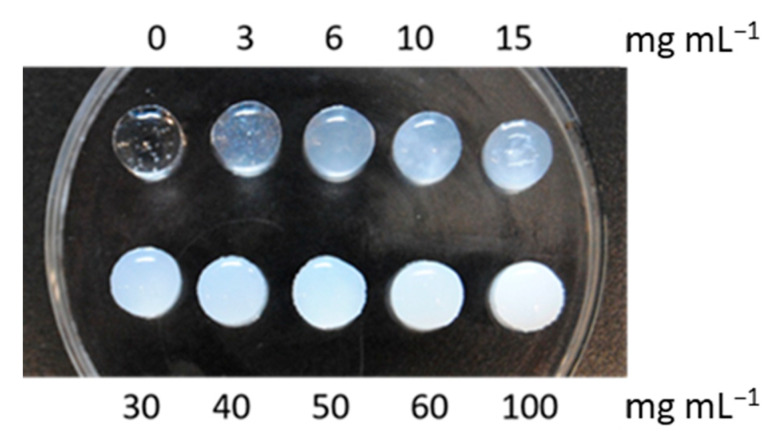
Hyaluronic acid gels (0.5 mL) with different concentrations of calcined mesoporous silica nanoparticles. The HA-based gels were able to tolerate very high loading of nanoparticles (up to 100 mg mL^−1^).

In order to study the capability of in situ-forming gels to encapsulate nanoparticles, a series of hyaluronic acid gels were first prepared with different amounts of calcinated MSNs. As shown in Figure 4, even with very high amounts of MSNs (up to 100 mg mL^−1^) added to the hyaluronic acid precursor, gels could be formed. In further experiments, an intermediate amount of MSNs (6 mg mL^−1^) was incorporated into HA gels and chitosan/β-glycerophosphate gels.

**Figure 5:**
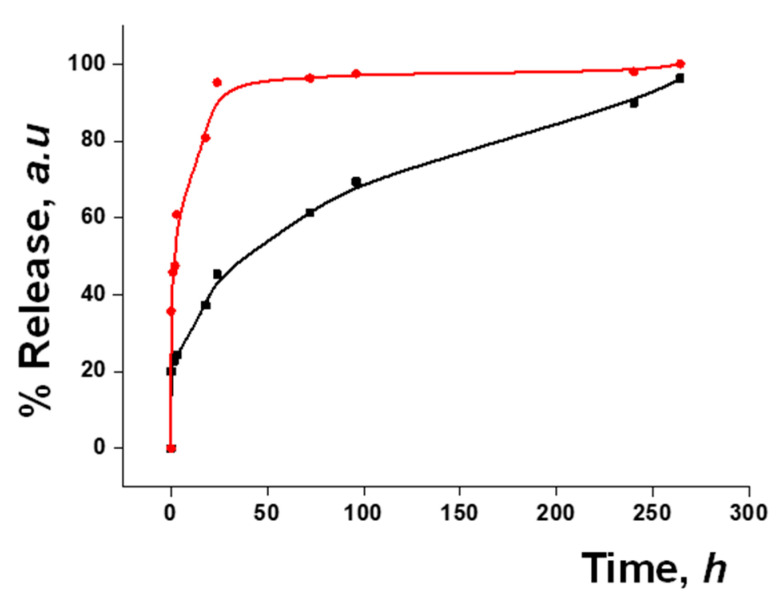
Release profile of MSNs as quantified by NTA from HA-based (black line) and chitosan-β-GP (red line) gels in PBS.

In order to evaluate and compare the nanoparticles’ release from the different in situ-forming matrices, gels were incubated with PBS at 37 °C while shaking at 100 rpm. Aliquots were removed at certain time points, and the volume was replaced with fresh pre-warmed PBS. All release samples were analyzed by NTA in order to quantify the amount of MSNs that escaped from the gels as a function of time. As displayed in Figure 5, a more sustained release of nanoparticles was noticed from the HA gel as compared to the chitosan/β-GP gel. A fast release of nanoparticles was observed from the chitosan-β-GP gel, achieving 80% of the maximum release of the entrapped MSNs in less than 48 h. In contrast, in the case of the HA-based gel, only around 50% of the nanoparticles were released after 48 h. After 10 days, a maximum delivery of nanoparticles from both gels was observed. Due to the slower release profile of the entrapped MSNs from the HA-based gel, this candidate was selected for further studies.

### 3.5. Gated Response of Nanoparticles Inside the HA Gel Matrix

**Figure 6:**
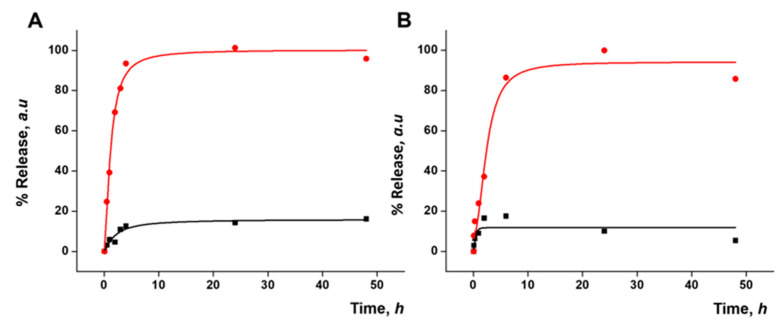
Kinetic release profile of safranin O delivered from HA gel containing 6 mg·mL^−1^ of S2 (**A**) or of DOX delivered from HA gel containing 6 mg·mL^−1^ of S3 (**B**) in the absence (black) and in the presence (red) of GSH.

As a step forward, the response of the prepared gated materials encapsulated in the gel in the presence of a redox stimulus was evaluated. As in the previous results, HA gels with S2 (HA-S2) or S3 (HA-S3) nanoparticles at a concentration of 6 mg mL^−1^ were used. The final HA-S2 and HA-S3 gels were placed in a well plate both in the absence and presence of 10 mM GSH. Payload release was monitored by measuring the fluorescence signal of the cargo molecules as explained above. Figure 6 shows the release profiles of safranin O (A) and DOX (B) from the corresponding gels up to 48 h. For HA-S2, a massive release of safranin O was observed in the presence of GSH, whereas a poor release was observed in its absence. A similar outcome was observed in the case of HA-S3 for DOX release. Both release profiles demonstrated that the gated materials keep their cargo-delivery-responsive behavior after being encapsulated in the gel. Cargo release is due to the reduction in the disulfide bonds in the capping ensemble on the nanoparticles that induces pore opening. Taking into account the delivery profile of the nanoparticles from the gel (Figure 5), the cargo delivery from the nanoparticle alone in the presence of GSH (Figure 3), and the cargo delivery from the nanoparticle in the HA gel in the presence of GSH (Figure 6), it seems that the delivery of both safranin O and DOX from the S2 and S3 materials loaded in the gel is slower than the release of the same cargo from the free nanoparticles, probably due to a delayed diffusion of the trigger (GSH) into the gel structure. Cargo delivery from the nanoparticles embedded in the HA gel in the presence of GSH most likely happens in two ways: partially from nanoparticles that have been released from the gel and partially from nanoparticles that are still encapsulated in it, reached by GSH diffusion.

In addition, in order to confirm the integrity of the nanoparticles released from the HA-based gels, degradation studies were carried out over a period of 10 days. HA gels containing S2 or S3 at a concentration of 6 mg mL^−1^ (HA-S2 and HA-S3) were prepared as described above. Subsequently, HA-S2 and HA-S3 gels were added to a 12-well plate and incubated at 37 °C with continuous shaking. The media were removed daily and replaced with fresh pre-warmed media. In an attempt to remove possible products of gel degradation, the release media were centrifuged at 2,000 rpm for 2 min. The supernatant was measured using DLS and TEM. The TEM images of day 10 samples from HA-S2 and HA-S3 (Figure S3A,B, respectively) show that S2 and S3 maintain their spherical morphology and size after the loading and release process. Furthermore, the internal structure is also preserved since it is possible to distinguish the typical pore channels of the mesoporous matrix. Additionally, analyzing the released particles by DLS, we observed different populations (table in Figure S3C): while the smaller ones were attributed to gel degradation products that were not completely removed by the centrifugation step, the size of the particles released from both HA-S2 and HA-S3 were in the range of the original S2 and S3 materials.

### 3.6. Cellular Studies

**Figure 7:**
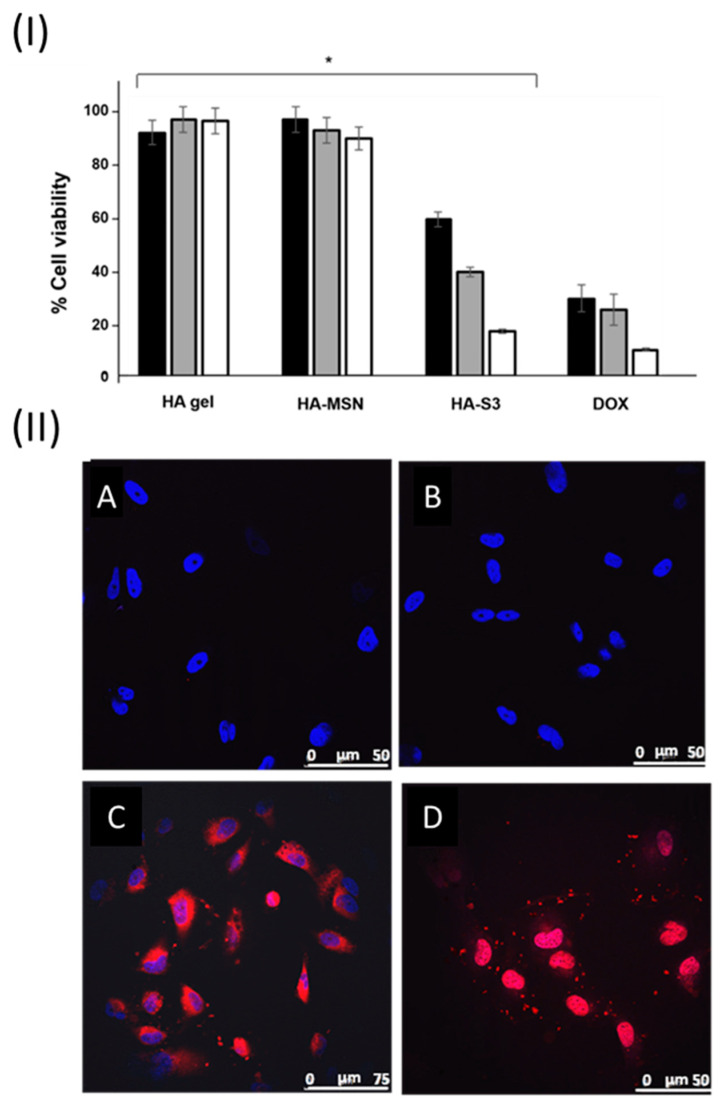
(**I**) Quantification of viable cells as measured by WST-1 assay at 24 h (black), 48 h (grey), and 72 h (white bar). Three independent experiments containing triplicates were performed. Statistically significant differences were observed among the cells treated with HA-S3 when compared to cells treated with the control HA gel or HA-MSN (Student’s *t* test, * = *p* < 0.05). The results are expressed as mean ± se. (**II**) Confocal microscope images corresponding to U271 cells treated for 24 h with HA gel (**A**), HA-MSN (**B**), HA-S2 (**C**), and HA-S3 (**D**) loaded with 6 mg mL^−1^ of the corresponding nanoparticles.

The mechanism of action of DOX determines both drug intercalation into the DNA strands and the disruption of the topoisomerase-II-mediated DNA repair, as well as the generation of free radicals with consequent damage to cellular membranes, DNA, and proteins [36]. According to this, a decrease in cell viability is expected as a result of the treatment. In order to assess the bioactivity of the prepared HA gel formulations, U271 cells were cultured in adherent 24-well plates at a density of 1.5 × 10^4^ cells/well in fully supplemented MEM growth media, under normal culture conditions. After 24 h, 900 µL of medium was replaced by fresh medium in each well, and a Falcon culture insert with an 8.0 µm pore size PET membrane was gently placed into the well. Then, 350 µL of pre-warmed medium was added to the insert before different HA gel formulations, previously sterilized under a UV lamp for 20 min, were placed inside. Specifically, HA (gel without nanoparticles), HA-MSN (gel with bare MSNs), and HA-S3 (gel containing S3) were tested in different inserts, and cell viability was measured after 24, 48, and 72 h using WST-1 (see Figure 7I). For comparison, the viability of cells treated with free DOX at the same concentrations as those released by HA-S3 in PBS with 10 mM GSH (3.79, 3.93, and 5.71 µg of DOX after 24, 48, and 72 h, respectively) was determined.

Quantification of cell viability showed no important toxicity for HA and HA-MSN after 24, 48, and 72 h, as expected. In comparison, a decrease of about 40% in cell viability was found 24 h after treatment with HA-S3, when compared to controls. This drop was caused by the release of S3 nanoparticles from the HA-S3 gel, their crossing of the inset membrane, the subsequent S3 uptake by the cells, and the final DOX release inside the cells thanks to cytosolic GSH. At longer time points, the viability percentage decreased, reaching values of around 20% at 72 h. A similar trend for the free DOX was observed. The modest differences in percentage values between HA-S3 and free DOX are probably due to the longer time that S3 nanoparticles need to escape from the gel and reach the cells, in comparison with the quick free DOX uptake. These results support our hypothesis of a sustained release of the nanoparticles from the gel and further DOX release inside the cells with the subsequent reduction in cell viability. Thus, our work demonstrates that HA-S3 and similar systems represent a suitable strategy to deliver gated nanoparticles locally.

Moreover, confocal microscopy assays using cell culture inserts with HA, HA-MSN, HA-S2, and HA-S3 were performed to check the cellular internalization of the nanoparticles delivered from the gels and their triggered release in U217 cells. Cells were also stained with the DNA marker DAPI. The obtained results after 24 h are shown in Figure 7II. As expected, the only fluorescent signal in the controls, HA and HA-MSN, comes from the nuclei staining (blue), whereas a perinuclear red signal is observed in the images for cells treated with HA-S2 and HA-S3. In Figure 7(IIC), the red fluorescence was assigned to an accurate release of S2 material from the HA-S2 gel formulation, the uptake of the material by U271 cells with subsequent cleavage of the disulfide bonds of the PEG cap, and safranin O release. Similar results were observed for S3 material from the HA-S3 formulation (Figure 7(IID)). The released S3 nanoparticles from HA-S3 were also successfully internalized by the cells and the DOX delivered. In addition, cells treated with HA-S3 presented a cell death phenotype compared to control samples and HA-S2. These microscopy images demonstrate the intracellular localization of nanoparticles previously delivered from the gels and the subsequent release of safranin O and DOX.

There are a limited number of examples of stimuli-responsive depots, and, in most of these approaches, the polymer matrix responds to stimuli such as temperature [37,38], pH [39,40], the presence of enzymes [41], electric fields [42], etc., and the cargo release is controlled by changes induced in the stability or conformation of the polymer or by the cleavage of certain chemical bonds. In contrast, our system is designed to contain gated nanoparticles that are sustainably delivered from the depot as a function of time. Our studies demonstrate that, in the presence of a redox stimulus (i.e., GSH), drug delivery from the depot is observed. Most interestingly, we have also found that the depot can serve as a suitable container for gated nanoparticles that can be released from the matrix to the media and further internalized into the cells, where the drug is finally delivered due to the high concentration of cytosolic GSH. The encapsulation of gated materials confers our system a competitive advantage in developing local sustained nanoparticle delivery systems able to release an encapsulated drug over extended periods in response to biological stimuli.

**Figure 8:**
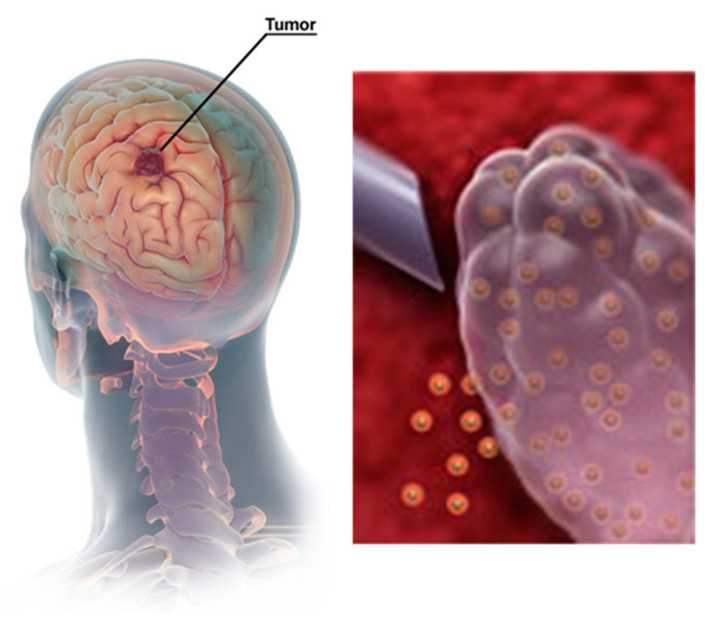
Schematic representation of stimuli-responsive drug nanocarriers locally released from a gel depot. In situ-forming gels may be injected in the tumor environment in order to facilitate a sustained release of the nanoparticles upon degradation.

In the context of malignant gliomas, an exciting purpose of such combination strategies is the implantation of hydrogels containing drug-loaded MSNs in the tumor environment to shrink the size of the lesion in hard-to-reach areas or, following the surgical removal of the tumor, to prevent metastasis or cancer recurrence (Figure 8). Surgery, indeed, rarely removes all tumor cells in glioma because of their invasive nature; hence, postoperative treatment is frequently required [43]. One advantage of local delivery is that the therapeutic dose can be maintained at the tumor site. On the other hand, the MSNs guarantee a continuous and extended delivery of drugs to the surviving malignant cells. As a result, the treatment of GBM using this combination is highly promising.

In summary, it has been demonstrated that the combination of in situ-forming gels with responsive mesoporous silica nanoparticles is a suitable strategy to design carriers for the local sustained release of chemotherapeutics. The gel matrix provides a progressive release of drug-loaded nanoparticles that can subsequently deliver the drug after cell internalization. Specifically, mesoporous nanoparticles were successfully loaded with a fluorescent dye (safranin O) or with a chemotherapeutic agent (DOX), and then capped with a disulfide-containing polyethylene glycol derivative acting as a molecular gate on the surface of the nanoparticle. Release studies carried out with the nanoparticles demonstrated that payload delivery is triggered in the presence of the reducing agent, GSH, due to the cleavage of the disulfide bonds that induced pore opening. It was also found that the nanoparticles remained stable after their encapsulation in the gel network. Finally, we also demonstrated that HA depots loaded with gated nanoparticles containing DOX are able to deliver nanoparticles to the medium which are then internalized into U271 cells (a malignant glioma cell line), where the gating ensemble is open due to the high concentration of cytosolic GSH, and DOX is delivered, significantly reducing cell viability. We believe that this or similar systems open the way to multiple possibilities for combining the tunable properties of the gels with a wide range of stimuli-responsive nanoparticles, with the aim of improving the targeted controlled release of chemotherapeutics for certain applications, such as gel implantation in the tumor environment.

## Figures and Tables

**Table 1:** BET specific surface values, pore volumes, and pore sizes calculated.

Solid	SBET (m^2^ g^−1^)	Total Pore Volume ^1^ (cm^3^ g^−1^)	Pore Size ^1,2^ (nm)
**MSNs**	919.62	0.91	3.22
**S1**	475.18	0.43	-
**S2**	102.01	0.24	-

^1^ Pore volumes and pore sizes were associated with only intraparticle mesopores. ^2^ Pore size estimated by the BJH model applied to the adsorption branch of the isotherm.

**Table 2:** Content (α) of anchored molecules and cargo in millimoles per gram of SiO_2_ for solids S2 and S3.

Solid	^α^Saf O (mmol g^−1^ SiO_2_)	^α^DOX (mmol g^−1^ SiO_2_)	^α^PEG (mmol g^−1^ SiO_2_)
**S2**	0.24	-	0.49
**S3**	-	0.21	0.35

## Data Availability

Data is contained within the article.

## Supplementary Materials

The following supporting information can be downloaded at: https://www.mdpi.com/article/10.3390/pharmaceutics15041071/s1, Figure S1. Size distribution by number of particles obtained by DLS studies for MSN, S2 and S3; Figure S2. Nitrogen adsorption-desorption isotherms for calcined MSNs (black), S1 material (red) and S2 material (blue). The inset shows the pore size distribution of the starting MSNs; Figure S3. (A) TEM images of nanoparticles released from HA-S2 gels after 10 days. (B) TEM images of nanoparticles delivered from HA-S3 gels after 10 days. (C) Mean size values from DLS studies of nanoparticles after 10 days from HA-S2 (A) and HA-S3 (B).
