# Supplementary material for: In Situ-Forming Gels Loaded with Stimuli-Responsive Gated Mesoporous Silica Nanoparticles for Local Sustained Drug Delivery
Source: Pharmaceutics. 2023 Mar 26;15(4):1071. doi: 10.3390/pharmaceutics15041071 (PMC10144720; doi:10.3390/pharmaceutics15041071)
Supplement: Supplementary file 1 [file pharmaceutics-15-01071-s001.zip › pharmaceutics-2216349-supplementary.pdf]

Supporting information

In Situ-Forming Gels Loaded with Stimuli-Responsive Gated Mesoporous Silica Nanoparticles for Local Sustained Drug Delivery

Cristina de la Torre [1,2], Carmen Coll [3], Amelia Ultimo [3,*], Félix Sancenón [1,2,4], Ramón Martínez-Máñez [1,2,4] and Eduardo Ruiz-Hernández [3,*]

[1] *Instituto Interuniversitario de Investigación de Reconocimiento Molecular y Desarrollo Tecnológico (IDM), Universitat Politècnica de València, Universitat de València, Camino de Vera s/n, 46022 Valencia, Spain*

2 *CIBER de Bioingeniería, Biomateriales y Nanomedicina (CIBER-BBN), 28029 Madrid, Spain*

3 *School of Pharmacy and Pharmaceutical Sciences, Trinity College Dublin (TCD), D02 W272 Dublin, Ireland*

4 *Departamento de Química, Universitat Politècnica de València, Camino de Vera s/n, 46022 Valencia, Spain*

<u>Materials characterization</u>

DLS studies

Dynamic light scattering (DLS) studies for **MSNs** calcinated, **S2** and **S3** were performed. All measurements were carried out at 25 °C using a Malvern Zetasizer Nano ZS instrument in triplicate, on previously sonicated dispersions of the nanomaterials in water at concentration of 0.01 mg mL^{-1}. Results are listed in Figure S1. As can be seen, an increase in the hydrodynamic particle diameter after the surface modification was observed, which resulted in a diameter of 122.5 nm and 140.2 for the final **S2** and **S3** nanodevices, respectively.

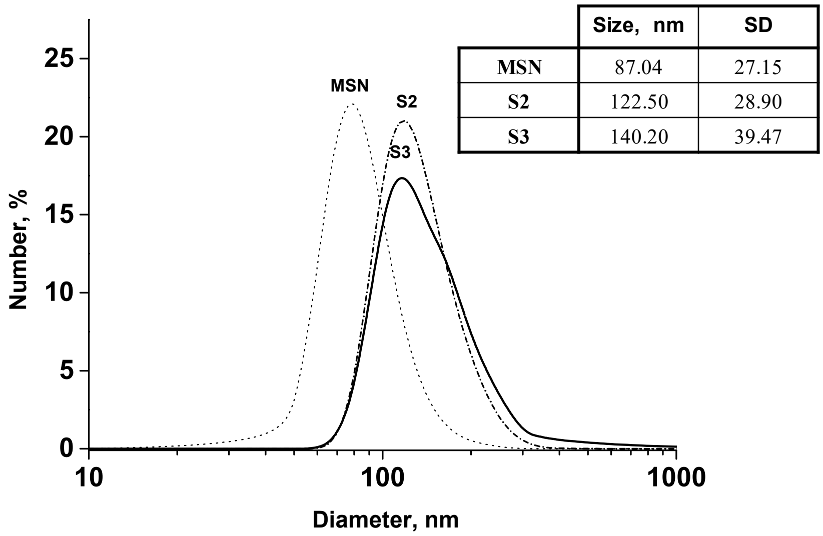

	Size, nm	SD
MSN	87.04	27.15
S2	122.50	28.90
S3	140.20	39.47

Figure S1. Size distribution by number of particles obtained by DLS studies for MSN, **S2** and **S3**.

N_2 adsorption-desorption isotherms

The N_2 adsorption-desorption isotherms of the calcinated MSN material showed two adsorption steps typical of these mesoporous materials (black curve in Figure S2). A first adsorption step at P/P_0 values between 0.1 and 0.3, in agreement with a type IV isotherm corresponding to the nitrogen condensation inside the mesopores by capillarity, was observed. With the application of Barret-Joyner-Halenda (BJH) model on the adsorption branch of the isotherm and PXRD studies, a pore diameter and a pore volume were calculated to be 3.2 nm and 0.91 $cm^3 g^{-1}$, respectively. Using the Brunauer, Emmett and Teller (BET) model, a specific surface area of 919.6 $m^2 g^{-1}$ was calculated. A second feature of the N_2 adsorption-desorption isotherms of the calcined MSN appeared on the curve at high relative pressure (P/P0 > 0.8), which corresponds to the filling of the large pores between the nanoparticles due to textural porosity. On the other hand, N_2 adsorption-desorption isotherms of **S1** and **S2** were typical of mesoporous with filled mesopores (see Figure S2 red and blue curves, respectively).

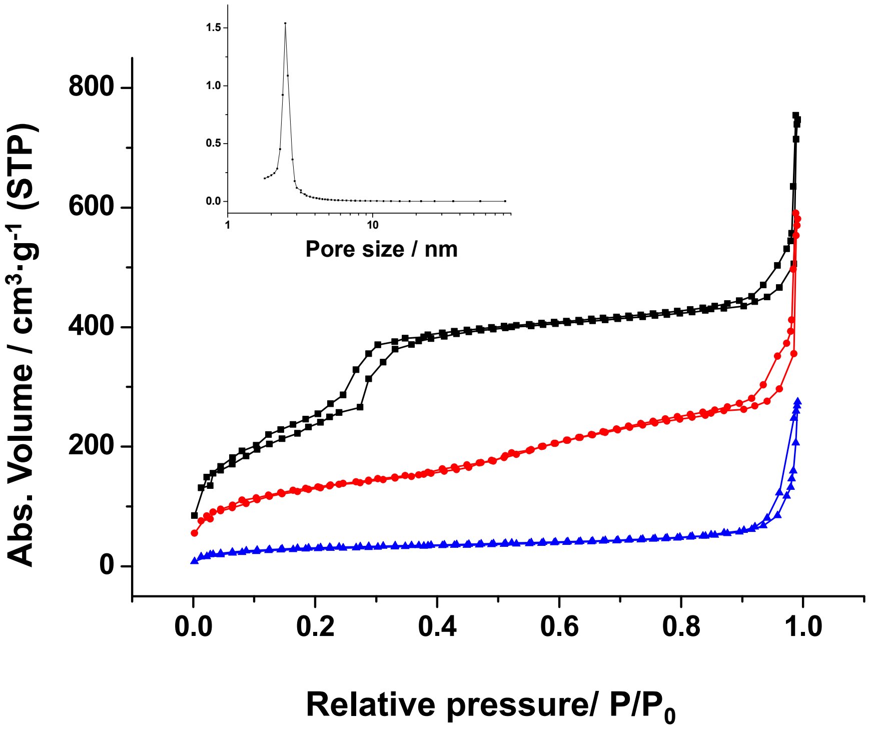

Figure S2. Nitrogen adsorption-desorption isotherms for calcined MSNs (black), **S1** material (red) and **S2** material (blue). The inset shows the pore size distribution of the starting MSNs.

Degradation of HA gels loaded with silica nanoparticles

A)

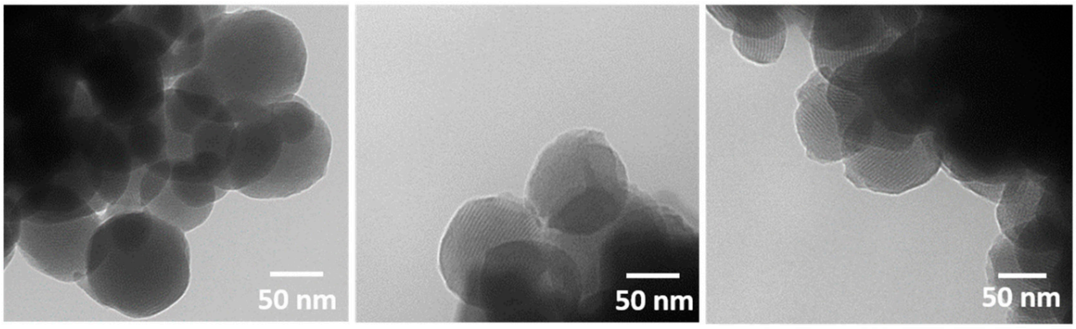

B)

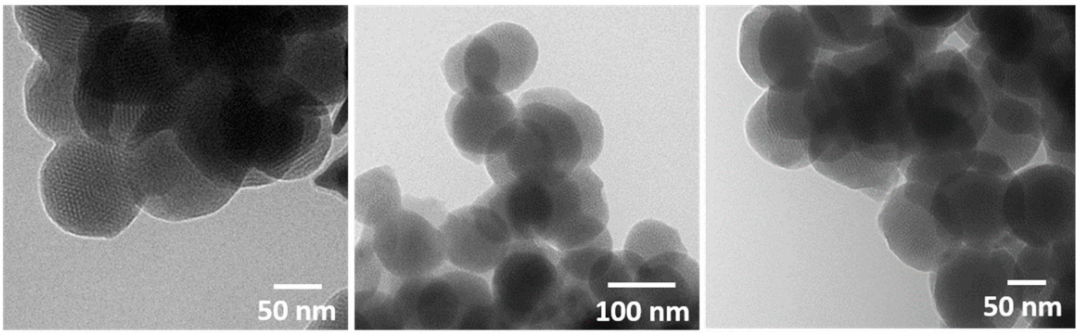

C)

	Diameter (nm)	% of particles (Intensity)
A	1.6 ± 7.2	20
	26.6 ± 8.5	54
	157.1 ± 47.9	20
B	1.1 ± 7.9	17
	27.4 ± 5.9	45
	189.0 ± 48.1	33

Figure S3. (A) TEM images of nanoparticles released from **HA-S2** gels after 10 days. (B) TEM images of nanoparticles delivered from **HA-S3** gels after 10 days. (C) Mean size values from DLS studies of nanoparticles after 10 days from **HA-S2** (A) and **HA-S3** (B).
